# Serial MRIs provide novel insight into natural history of optic pathway gliomas in patients with neurofibromatosis 1

**DOI:** 10.1186/s13023-018-0811-9

**Published:** 2018-04-23

**Authors:** Laura Sellmer, Said Farschtschi, Marco Marangoni, Manraj K. S. Heran, Patricia Birch, Ralph Wenzel, Victor-Felix Mautner, Jan M. Friedman

**Affiliations:** 1 Department of Medical Genetics, BC Children’s Hospital, University of British Columbia, 4480 Oak Street, Vancouver, Canada; 2 Department of Neurology, University Hospital Hamburg-Eppendorf, Hamburg, Germany; 3 Diagnostic and Therapeutic Neuroradiology, University of British Columbia, Vancouver, Canada; 4 Department of Radiology, MRI Institute Altona, Hamburg, Germany

**Keywords:** Optic pathway glioma, Neurofibromatosis 1, Cohort study, Glioma, Adults, Children

## Abstract

**Background:**

Optic pathway gliomas (OPGs) are present in 20% of children with neurofibromatosis 1 (NF1) but are less frequently observed in adults. Our goal was to determine the natural history of OPGs in children and adults with NF1.

**Results:**

We analyzed the features of OPGs and other intracranial lesions on 1775 head MRI scans of 562 unselected adults and children with NF1 collected between 2003 and 2015. 52 (9.3%) of 562 patients in this study had an OPG diagnosed on their MRI. The median age at first scan with an OPG present was 12.7 years. Of the 52 OPG patients, the intraorbital optic nerves were affected in 29 patients (56%), the prechiasmatic optic nerves were affected in 32 patients (62%), the optic chiasm was affected in 17 patients (33%) and the optic radiations were affected in 19 patients (37%). 29 patients had two or more areas affected. One patient had a newly-appearing OPG, and 1 patient showed progression. The rate of progression over 5 years was 2.4% (95% CI: 0.4% to 16%). Four patients showed partial regression of their OPGs, but we observed no case of complete regression during this study. The rate of regression over 5 years was 8.9% (95% confidence intervals: 2.8% to 26%). We found the presence of UBOs and the presence of OPGs in individual patients to be highly associated (*p* = 0.0061).

**Conclusion:**

OPGs are more common in older adults with NF1 than previously thought. The occurrences of unidentified bright objects (UBOs) and asymptomatic OPGs are associated with each other. This suggests the possibility that OPGs that remain asymptomatic may differ pathogenically from those that become symptomatic.

**Electronic supplementary material:**

The online version of this article (10.1186/s13023-018-0811-9) contains supplementary material, which is available to authorized users.

## Background

NF1 is a dominantly inherited multisystem disorder affecting 1 in 3500 individuals [1]. It is caused by mutations in *NF1*, a large gene located on the long arm of chromosome 17 [2–4]. NF1 is a neurocutaneous disorder characterized by the development of dermal and plexiform neurofibromas and café-au-lait spots [5]. One of the most serious manifestations of NF1 in children is the development of optic pathway gliomas (OPGs). These tumours affect approximately 20% of all children with NF1 [6] and can lead to a loss of vision, proptosis, or precocious puberty. Fortunately, however, these tumours remain asymptomatic in the majority of affected children. It is currently not recommended to screen children with NF1 routinely by MRI for optic pathway gliomas, as the vast majority of tumours are indolent, and early detection does not improve visual outcomes [7].

Factors such as tumour location [8] and changes in tissue microstructure [9] have been proposed to predict which OPGs will become symptomatic; however, these findings remain controversial. This is largely because the natural history of OPGs in people with NF1 has not been thoroughly characterized: There are no large studies of adult NF1 patients with OPGs, and we do not even know the prevalence of this tumour in adults with NF1.

In this study, we used routine MRIs to investigate the prevalence and natural history of optic pathway gliomas in children and adults with NF1.

## Methods

### Patients

All NF1 patients seen in the NF outpatient department of the University Hospital Hamburg-Eppendorf between 2003 and 2015 were offered whole-body and head MRIs as part of a routine tumour monitoring protocol [10]. Since MRIs were offered to all patients independent of their clinical symptoms, the images are representative of the patient population seen in the clinic. Informed consent was obtained from all subjects, and the ethical committees of the Medical Chamber of Hamburg and the University of British Columbia approved the study.

### Magnetic resonance imaging (MRI)

To evaluate the extent of optic gliomas, we defined four locations: intraorbital optic nerves, prechiasmatic optic nerves, chiasm, and optic tracts/optic radiations. A glioma of the optic pathway was diagnosed if there was hyperintensity on T_2_-weighted images or if they enhanced after contrast injection. A tumour was defined as being multifocal of it was present in two or more unconnected sections of the optic pathway.

Based on the 1775 clinical MRI reports, a list of patients was generated who had been clinically diagnosed with OPG. All head MRIs from these patients were re-evaluated by two neuroradiologists in Canada (M.M. and M.K.S.H.), and the presence of OPGs in each individual MRI study was established by consensus using the criteria described above.

### Features extracted from MRIs and clinical records

Clinical features extracted from the MRI reports of all 562 patients included: Presence of OPG, presence of non-optic gliomas, presence of unidentified bright objects (UBOs) and presence of plexiform neurofibromas (PNs) on the corresponding whole-body MRI examination. The presence of subcutaneous neurofibromas in patients with OPG was determined from the corresponding whole-body MRI.

The last visual acuity measurements during the study period were used to determine the presence or absence of loss of vision and visual field defects.

### Descriptive statistical analysis

Patients were divided into 10-year age groups and counted only once per age group. 95% confidence intervals of OPG prevalence were calculated as ±1.96 standard deviations of a binomial distribution.

Multiple logistic or linear regression was performed to identify factors associated with OPG presence or volume. Natural log transformation was applied to the tumour volumes to achieve normal distribution for linear regression. Predictor variables for logistic regression with presence of OPG as the outcome variable were age at first scan with OPG present and the presence of non-optic gliomas, UBOs, or plexiform neurofibromas. Predictor variables for analysis of OPG volume were age at first scan with OPG present, and the presence of non-optic gliomas, UBOs, plexiform neurofibromas or subcutaneous neurofibromas.

We used the Kaplan-Meier method to calculate the cumulative rates and 95% confidence intervals of OPG progression or regression within 5 years of first MRI diagnosis. The log-rank test was used to assess differences between the rates of progression or regression in patients under 20 years of age and those over 20 years of age. These analyses were performed with IBM SPSS Statistics version 24.

UBO prevalence in patients with or without OPGs was compared using the Mantel-Haenszel test. Each patient was counted once after stratification into a single age group for this analysis: 0–9.99, 10.0–19.99, 20.0–29.99, 30.0–39.99 or ≥ 40 years old at the time of first MRI or first MRI on which an OPG was seen in the study. The calculation was performed using IBM SPSS Statistics version 24.

Results with *p* ≤ 0.05 were considered to be statistically significant.

## Results

### Demographics

562 NF1 patients (264 males and 298 females) were included in this study. A total of 1775 whole-body and head MRI examinations were performed on these patients between 2003 and 2015, with a median follow-up time of 3.7 years (range 0 to 13.0 years) and a median number of 3 scans per person (range 1 to 13 scans). At the time of the MRI scan, patient ages ranged from 0.4 to 72.8 years. During the study, 75 patients were lost to follow-up or died of a reason unrelated to OPG, equaling a dropout rate of 13.3%.

### Prevalence of optic pathway glioma per age group

**Fig. 1:**
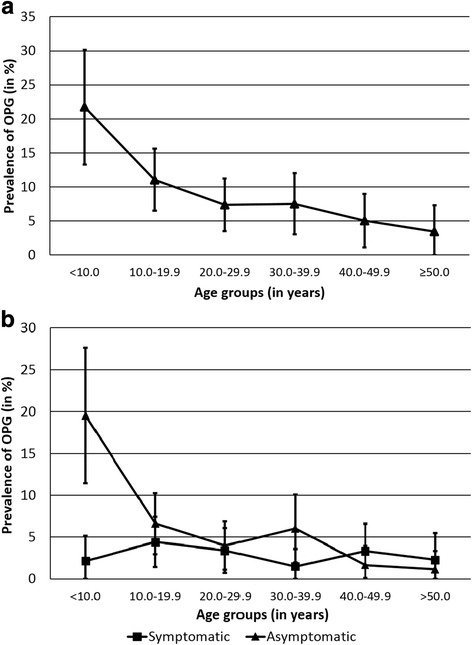
Prevalence of optic glioma per age group. **a** Total prevalence of OPG per age group. Error bars are 95% confidence intervals of a binomial distribution. **b** Prevalence of asymptomatic and symptomatic OPGs per age group. Error bars are 95% confidence intervals of a binomial distribution

56 patients received a clinical diagnosis of OPG based on their brain MRIs. In 52 patients, two independent neuroradiologists confirmed the diagnosis of OPG using the study criteria (see Table 1 and Additional file 1: Table S1). The overall prevalence of OPG among NF1 patients in this study was 9.3%. The prevalence was highest among children aged 10 or younger and declined with advancing age (Fig. 1a). The median follow-up of all NF1 patients who had OPGs was 5.2 years (range: 0–13.0 years), with a total of 283.2 patient years of follow-up.

**Table 1:** Features of symptomatic optic pathway gliomas in 17 of 52 OPG patients. OPG location indicates extent after shrinkage in patients with regression or extent after growth in patients with progression of the tumour. Details on asymptomatic OPG patients can be found in Additional file 1: Table S1

Patient number	Sex	Age at first scan (years)	OPG location	Enhancement	Changes during follow-up	Symptoms	Age at symptom onset (In years)	Treatment	Changes after treatment
4	F	19.7	Left intraorbital ON	No	Stable	*Reduced vision in left eye*	*3*	–	–
5	F	10.8	Chiasm	Avid	Stable	*Reduced vision and visual field defects in both eyes*	*11*	Vincristin, carboplatine	Tumour shrinkage and improvement of vision
13	M	6.2	Right prechiasmatic ON, chiasm, right radiations	Avid	Stable	*Reduced vision in both eyes, visual field defect in right eye, premature puberty*	*6*	Vincristin, carboplatine, leuprorelin	Improved vision
14	M	25	Right and left intraorbital ON	No	Stable	*Reduced vision in left eye, visual field defect in left eye, hydrocephalus*	*4*	–	–
17	F	8.7	Right and left intraorbital ON	No	Stable	*Reduced vision in both eyes*	*5*	Vincristin, carboplatine	Improved vision
18	M	30.3	Right and left prechiasmatic ON, chiasm, right and left radiations	Diffuse	Stable	*Reduced vision in right and left eye, visual field defects in both eyes*	*6*	–	–
29	M	18.1	Left prechiasmatic ON, chiasm, right and left radiations	Mild	Stable	*Blind in left eye, reduced vision and visuals field defect in right eye*	*6*	–	–
30	F	15.3	Right and left intraorbital ON, right and, left prechiasmatic ON, chiasm, right and left radiations	Diffuse	Stable	*Reduced vision in both eyes, visual field defect in left eye*	*Unknown*	Vincristin, carboplatine	Decreased vision
31	M	48.9	Right and left intraorbital ON, right and left prechiasmatic ON	No contrast used	Stable	*Blind in both eyes*	*1.5*	–	–
33	F	34.6	Right prechiasmatic ON, chiasm	No	Stable	*Reduced vision in right eye*	*3*	–	–
35	F	25.4	Right intraorbital ON, right prechiasmatic ON, chiasm, right radiations	Mild	Stable	*Blind in right eye, reduced vision and visual field defect in left eye*	*4*	–	–
39	F	15.1	Left prechiasmatic ON, chiasm, left radiations	No contrast used	Stable	*Blind in left eye, reduced vision and visual field defect in right eye*	*11*	Radiation and surgery	Decreased vision
41	F	34.5	Left prechiasmatic ON	No	Stable	*Reduced vision in both eyes, visual field defects in both eyes, diffuse hydrocephalus*	*5*	–	–
43	M	12.3	Right and left intraorbital ON, right and left prechiasmatic ON, chiasm, right radiations	No	Stable	*Reduced vision in right and left eye, visual field defect in right eye, diffuse hydrocephalus*	*6*	Vincristin, carboplatine	Stable vision
45	M	46.1	Right intraorbital ON, right prechiasmatic ON, chiasm	No	Stable	*Reduced vision in both eyes, visual field defects in both eyes*	*4*	–	–
49	M	44.6	Left intraorbital ON	Avid	Stable	*Blind in left eye*	*34*	Surgery	Decreased vision
50	F	25.3	Right prechiasmatic ON	No	Stable	*Reduced vision in right eye*	*Unknown*	–	–

*Abbreviations*: *ON* Optic nerve

### Description of OPGs

An overview of the clinical features of all OPGs is presented in Table 1 (symptomatic patients) and Additional file 1: Table S1 (asymptomatic patients). Of the 48 gliomas that affected the optic nerves, 29 were unilateral and 19 were bilateral. 12 of 52 OPGs were multifocal, equaling a total number of 64 tumours. Tumour volumes per patient ranged from 95.4 mm^3^ to 9255 mm^3^, with a median tumour volume of 879 mm^3^. 45 of the 52 OPG patients had their MRI scans performed with contrast enhancement: 34 of these patients showed no enhancement, 2 patients showed diffuse enhancement, 3 patients showed mild enhancement and 6 patients showed avid enhancement of their tumours. The clinical head and whole-body MRI reports included in this study noted the presence of UBOs in 246 (43.8%) and plexiform neurofibromas in 321 (57.1%) patients. Subcutaneous neurofibromas were seen in 22 (42%) of the 52 NF1 patients with OPG (these tumours were only evaluated in patients with OPG).

17 of the 52 patients with OPGs were symptomatic. All experienced vision decline, while 13 also had visual field defects. Patients with symptomatic OPGs received one MRI per year, with ophthalmologic assessments in between. During those assessments visual acuity testing, visual field testing and occasionally OPG was performed. Of these 17 patients with symptomatic OPG, 7 were treated: Five received chemotherapy with vincristine and carboplatin, one underwent surgery, and one underwent surgery and radiation. Surgery was reserved for cases with pronounced visual decline and rapidly growing tumours. The radiation treatment performed on one patient took place in 1996 before the high risk of secondary malignancy in people with NF1 who are treated with radiotherapy was appreciated. One patient (Patient 13) experienced premature puberty and was treated with leuprorelin.

We investigated the prevalence of symptomatic and asymptomatic OPGs per 10-year age group (Fig. 1b). Asymptomatic OPGs are very prevalent in young children, and their frequency decreases with increasing age until adulthood. In contrast, the prevalence of symptomatic tumours remains stable throughout all age groups.

Non-optic gliomas were seen in 34 of the 562 NF1 patients included in this study: nine (17%) of the 52 patients with OPG but only 25 (5.15%) of 485 without OPG (*p* = 0.001).

### Newly-appearing OPGs

**Fig. 2:**
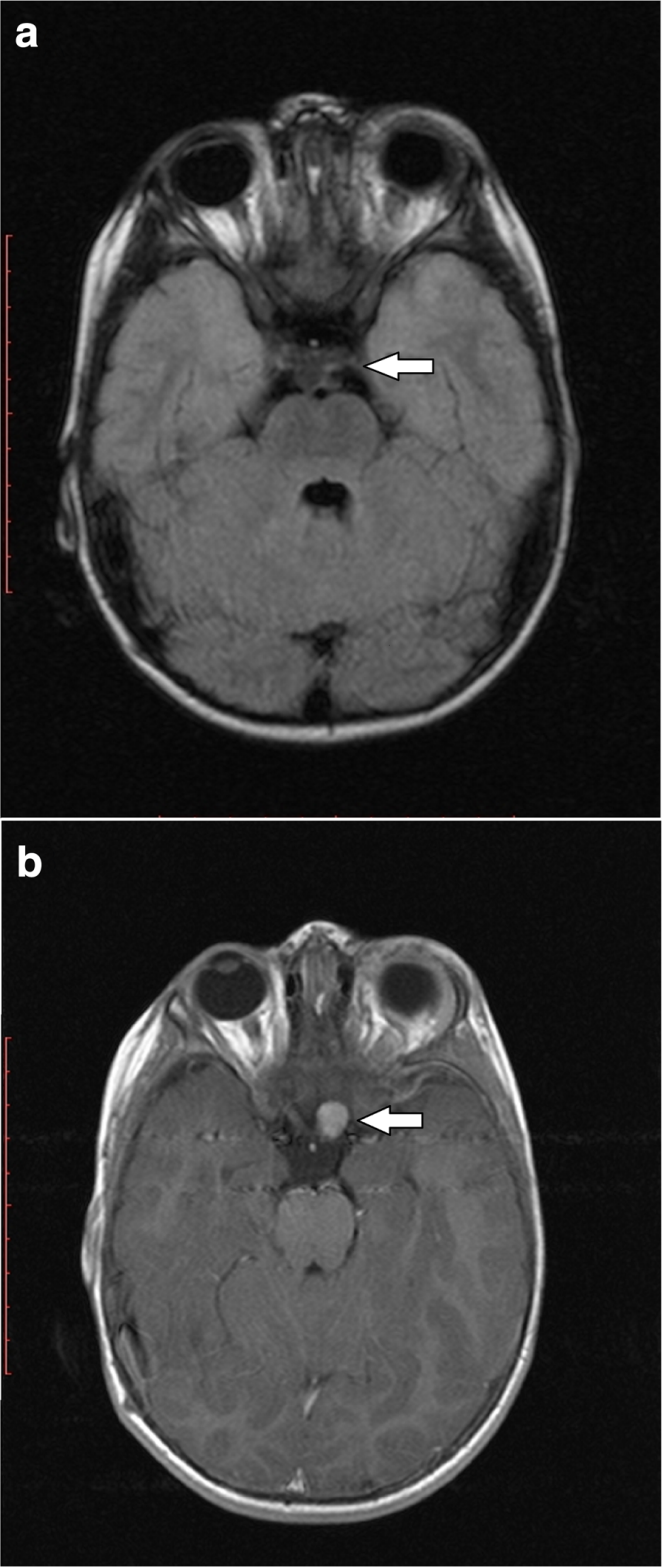
New appearance of an OPG in the left prechiasmatic optic nerve of Patient 8. **a** No glioma was seen at a scan performed when patient was 1.3 years old. No contrast matter was used in this scan. **b** An avidly enhancing left prechiasmatic optic nerve glioma with a volume of 1820 mm^**3**^ was apparent when the patient was 2.0 years of age. Both the left eye and the left intraorbital optic nerve were normal and well visualized in other image planes

There was only one patient (Patient 8) with an optic glioma that appeared during the course of this study (Fig. 2). After its first appearance at 2.0 years of age, the glioma was avidly enhancing. 3.5 years later the enhancement decreased from avid to diffuse. The patient remained asymptomatic throughout follow-up.

### OPG progression

**Fig. 3:**
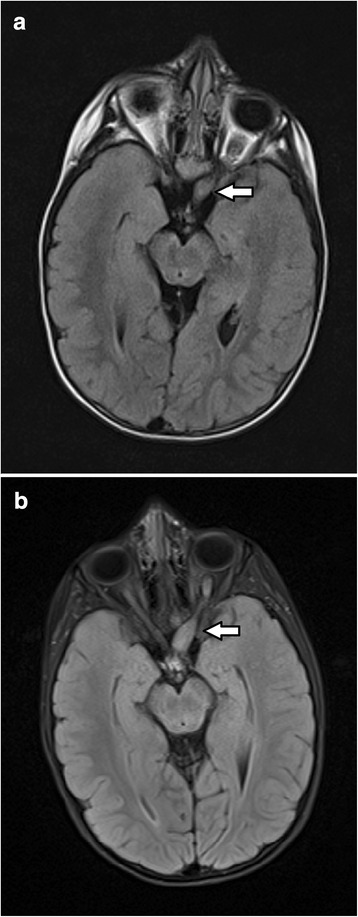
Progression of an OPG in Patient 37. **a** On the first scan performed at 4.0 years of age, a glioma measuring 1862 mm^**3**^ was present in the left intraorbital and prechiasmatic optic nerve. **b** On the next scan performed 1.0 years later, the OPG had increased in volume to 2636 mm^**3**^ and involved the optic chiasm as well as the left intraorbital and prechiasmatic optic nerve

Only one of the 52 NF1 patients with OPG (Patient 37) had a tumour that increased in volume during the study period (Fig. 3). The rate of progression was estimated as 2.4% over 5 years (95% confidence intervals: 0.4% to 16%). There was no significant difference in the cumulative rates of progression between children (< 20 years of age) and adults (≥20 years of age) (*p* = 0.42).

### OPG regression

**Fig. 4:**
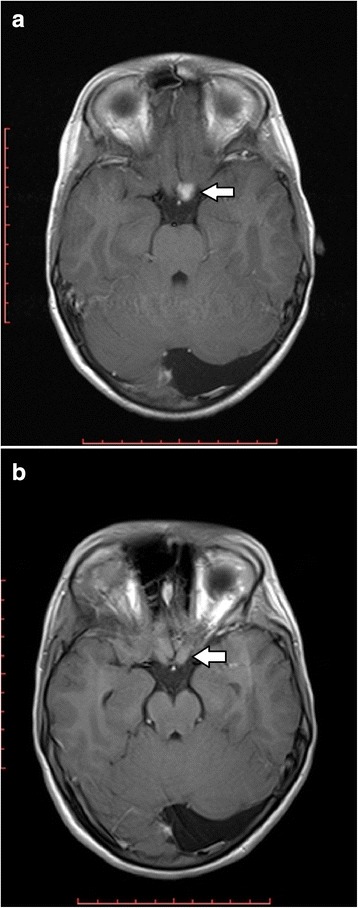
Spontaneous regression of an OPG in Patient 3. **a** A 516 mm^3^ left prechiasmatic optic nerve glioma on this patient’s first MRI at age 6.3 years. The tumour volume was unchanged over 2 MRI examinations during the next 2.2 years. **b** On re-examination at 9.3 years of age the OPG volume had decreased to 462 mm^3^, and the volume decreased again to 436 mm^3^ when the patient was 11.2 years old. The tumour volume was unchanged on the patient’s last MRI in this study at age 11.7 years

Four patients (Patients 3, 24 and 27) with OPG showed spontaneous shrinkage of their tumours during the period of observation (for Patient 3 see Fig. 4). The rate of regression was estimated as 8.9% over 5 years (95% confidence intervals: 2.8% to 26%). As with progression, there was no significant difference in the cumulative rates of regression between children and adults (*p* = 0.17). All instances of OPG regression occurred in patients under the age of 20 years, although only 54% of the patient-years of observation took place in this age group (*p* = 0.002). In at least three of these four patients (Patients 24, 27 and 48), the OPG showed enhancement prior to its shrinkage (no contrast enhanced studies were performed in Patient 3 after his first scan).

### Association of OPG presence and volume with other features observed on MRI examination

We performed multiple regression analysis to determine factors associated with OPG presence or volume in individual patients (see Additional file 2: Table S2). The presence of UBOs (OR = 2.4, 95% CI = 1.2 to 4.8) and the presence of non-optic gliomas (OR = 4.8, 95% CI = 2.0 to 12) each was associated with the presence of OPGs in a patient. Age at first scan was negatively associated with the presence of an OPG (OR = 0.96, 95% CI = 0.96 to 0.99). None of the independent variables significantly predicted tumour volume.

Because the prevalence of both UBOs and OPGs decreases with age [11], we investigated the relationship between UBOs and OPGs in individual NF1 patients after stratifying patients into 10-year age groups (Table 2). There is a strong overall association between the presence of OPGs and UBOs (Mantel-Haenszel summary odds ratio = 2.77, 95% confidence interval 1.39–5.53, *p* = 0.0061). This association was also seen in an analysis restricted to 34 patients with asymptomatic OPGs (Mantel-Haenszel summary odds ratio = 3.17, 95% confidence interval 1.29–7.80, *p* = 0.012).

**Table 2:** Frequency (and percentage) of UBOs among NF1 patients with or without OPG by 10-year age group

Age Group	OPG Absent	Any OPG Present	Asymptomatic OPG Present
0 to 9.99 years	53/72 (73.6%)	17/18 (94%)	16/17 (94%)
10.0 to 19.99 years	57/119 (47.9%)	10/13 (77%)	4/6 (67%)
20.0 to 29.99 years	39/109 (35.8%)	3/8 (38%)	3/5 (60%)
30.0 to 39.99 years	21/80 (26.3%)	3/8 (38%)	2/5 (40%)
40.0 years and above	43/129 (33.3%)	3/4 (75%)	1/1 (100%)

## Discussion

In this study we report the largest series of head MRIs described to date in unselected NF1 patients. Most previous studies have used convenience samples to estimate the prevalence of OPGs in children with NF1 [12, 13]. This approach, however, carries the inherent bias of the patients being selected for having clinical symptoms that required them to undergo imaging. In our study, every patient seen in the NF outpatient department was offered MRI, so our series is an unbiased representation of the patients seen in the clinic. Blanchard et al. recently performed a prospective head MRI study of 306 children with NF1 under 6 years of age and found the prevalence of OPG to be 14.7% (95% confidence interval: 11.0% to 19.3%), with 80% of patients being asymptomatic [13]. Other authors found similar prevalences in children, ranging from 15%–18% [12, 14, 15]. We found a somewhat higher prevalence (22%) among children with NF1 under 10 years of age: This might be due to different diagnostic criteria (T2 hyperintensity without consideration of thickness or tortuosity of the optic nerve) used in our study compared to the previous studies.

The prevalence of OPG in older children and adults with NF1 is lower than in young children but has rarely been evaluated. One retrospective study by Créange et al. found the prevalence in 138 individuals with NF1 over 18 years of age to be 5.8% [16]. In concordance with this finding, we found the prevalence in adults over 19.9 years of age to be 4.9% (95% confidence interval: 3.3% to 7.2%).

The decline in prevalence of OPG from childhood to adulthood might be explained in several different ways. Firstly, it is important to note that most OPGs are asymptomatic and are never confirmed by biopsy in people with NF1 [17, 18]. Optic nerve tortuosity and optic nerve sheath thickening are frequent in children with NF1 who do not have OPG [19], and it is not known if T2 hyperintensity or MRI enhancement of the optic nerves can occur in the absence of other evidence of neoplasia in this setting. Thus, it is possible that some OPGs diagnosed by MRI in children with NF1 are not true neoplasms.

Alternatively, there might be increased mortality in individuals with optic tumours, so that children with OPG are less likely to survive into adulthood. However, a study by Guillamo et al. showed that having an OPG is *not* a risk factor for premature death of NF1 patients [20].

It has been shown that having an OPG predisposes to the development of non-optic gliomas [21], which are associated with increased morbidity [20]. We found a strong correlation between presence of OPG and presence of non-optic gliomas: however, this cannot account for the strong decline of OPG prevalence with increasing age seen in our study, as no patients dies from non-optic glioma.

Lastly, tumours might regress spontaneously, as has been described many times for OPG in case reports of children with NF1 (see Additional file 3: Table S3 and [22–24]). Regression seems to occur mostly in tumours involving the optic chiasm, but may also sometimes occur in other sections of the optic pathway. In our study, we identified no instances of complete regression of an OPG but 4 cases of partial regression (see Additional file 1: Table S1). All of the patients in this study who showed tumour regression were under 20 years of age when this occurred. All three of the patients whose MRIs included contrast showed avid enhancement before regression and mild to no enhancement after regression had stopped.

Among the 17 symptomatic OPG patients, 7 were treated. Three of these 7 patients (Patients 5, 13 and 17) received chemotherapy soon after symptom onset, and all showed vision improvement after treatment. Of the remaining 4 patients, the age at symptom onset is unknown for 2 patients, 1 patient received surgery and radiation followed by a decline in vision, and 1 patient received chemotherapy 6 years after symptom onset, followed by stable vision. This may indicate a benefit to starting treatment early after symptom onset; however, our sample is too small to show any definitive benefit. Further research is required to investigate whether early treatment of symptomatic OPG is beneficial in NF1.

Sex has been suggested as a determinant of which NF1 OPGs become symptomatic. Females were reported to receive MRI for visual symptoms significantly more often than males and were 3 times more likely to undergo treatment for visual decline in one study [25] but not in another [13]. We did not see a difference in tumour location, tumour frequency symptom status or frequency of treatment initiation between males and females.

We observed a strong association between the presence of OPG and the presence of UBOs after stratification by age (Table 2); this association was also seen when the analysis was restricted to asymptomatic OPGs. Regression analysis showed a similar association between the presence of OPG and UBOs after adjustment for the effect of age (Additional file 2: Table S2).

There are several parallels between UBOs and asymptomatic OPGs: their glial origin, benign nature, usual spontaneous involution, frequent development in early childhood and very infrequent development later in life, and decreased prevalence with increasing age. UBOs are thought to be areas of immature myelin or intramyelinic edema [11, 26, 27] and *not* neoplasms. All studies investigating the histology of OPGs in NF1 patients only include symptomatic patients, as biopsy or surgical removal is not performed in asymptomatic patients. It has generally been assumed that the pathology (and pathogenesis) of symptomatic and asymptomatic OPG is the same in patients with NF1, but there is no direct evidence supporting this assumption. If many asymptomatic OPGs are actually areas of immature myelin instead of true neoplasms, it will change our understanding of NF1 pathology.

Recently, the idea that pediatric gliomas in general are neurodevelopmental disorders has gained traction. Pediatric gliomas vary from their adult counterparts in location (posterior fossa and optic pathway in children, supratentorial compartment in adults), their usual type (low-grade pilocytic astrocytoma in children, high-grade glioblastoma in adults) and their potential for malignant transformation (low in children, high in adults) [28]. OPGs in NF1 patients are often diagnosed in very young children, and few, if any, cases arise in adults. This stands in contrast to non-optic gliomas in NF1, which *do* often arise in adults [29, 30].

Our study has several limitations. First of all, our study population might not be representative of the NF1 population as a whole. We cannot rule out referral bias or the possibility that symptomatic patients are more likely to consent to participate in an MRI study than asymptomatic patients. Also, patients with more severe phenotypes are likely to receive more frequent clinical follow-up and repeat imaging than patient with less severe manifestations. Another important factor is the diagnosis of OPG based on imaging. There are no generally-accepted diagnostic guidelines for OPGs in NF1 patients, and diagnosis is based on clinical judgment.

## Conclusion

This is the largest prospective study of unselected head and whole-body MRIs ever performed in patients with NF1 [31]. It is also the first study that quantifies the frequency of OPG progression and regression. The observations that these lesions are extraordinarily frequent in children but are usually asymptomatic and remain so throughout their course and that many appear to regress spontaneously in late childhood or adolescence support our current clinical practice of only obtaining frequent follow-up MRIs in patients who have symptomatic OPGs or tumours that are growing or producing a mass effect. Our data are important for scientists working to understand the pathogenesis of NF1-associated lesions and for clinicians assessing NF1 patients who have OPG.

## Additional files

Additional file 1: **Table S1.** Overview of all 35 asymptomatic OPG patients. OPG location indicates extent after shrinkage in patients with regression or extent after growth in patients with progression of the tumour. None of the asymptomatic patients received any treatment for their OPGs. (DOCX 16 kb)

Additional file 2: **Table S2.** Age-adjusted associations of clinical features typical for NF1 with the presence of OPG. (XLSX 8 kb)

Additional file 3: **Table S3.** Published cases of spontaneous regression of symptomatic and asymptomatic OPG in NF1 patients. (XLSX 9 kb)
