# Supplementary material for: Serial MRIs provide novel insight into natural history of optic pathway gliomas in patients with neurofibromatosis 1
Source: Orphanet J Rare Dis. 2018 Apr 23;13:62. doi: 10.1186/s13023-018-0811-9 (PMC5913802; doi:10.1186/s13023-018-0811-9)
Supplement: Supplementary file 1 — Table S1. Overview of all 35 asymptomatic OPG patients. OPG location indicates extent after shrinkage in patients with regression or extent after growth in patients with progression of the tumour. None of the asymptomatic patients received any treatment for their OPGs. (DOCX 16 kb) [file 13023_2018_811_MOESM1_ESM.docx]

| **Patient number** | **Sex** | **Age at first scan (years)** | **OPG location** | **Enhancement** | **Changes during follow-up** | **Age at which change occurred (In years)** | **Age at symptom onset (In years)** |
| --- | --- | --- | --- | --- | --- | --- | --- |
| 1 | M | 32.1 | Left prechiasmatic ON | No | Stable | - | - |
| 2 | M | 5.2 | Chiasm, right radiations | No | Stable | *-* | *-* |
| 3 | M | 6.3 | Left prechiasmatic ON | No contrast used | Decreased in size | *7.3 & 11.3* | *-* |
| 6 | F | 23.1 | Left intraorbital ON | No | Stable | *-* | *-* |
| 7 | F | 5.1 | Right and left intraorbital ON | No | Stable | *-* | *-* |
| 8 | M | 1.3 | Left prechiasmatic ON | Avid | Newly-appearing | *2.0* | *-* |
| 9 | F | 54.1 | Right and left intraorbital ON, right and left prechiasmatic ON, chiasm | No | Stable | *-* | *-* |
| 10 | M | 2.7 | Right and left intraorbital ON | No contrast used | Stable | *-* | *-* |
| 11 | F | 10.8 | Right intraorbital ON | No | Stable | *-* | *-* |
| 12 | M | 10.2 | Right prechiasmatic ON | No | Stable | *-* | *-* |
| 15 | M | 11 | Right and left intraorbital ON | No | Stable | *-* | *-* |
| 16 | M | 2.4 | Left intraorbital ON, right prechiasmatic ON | No | Stable | *-* | *-* |
| 19 | M | 8.3 | Right and left intraorbital ON | No contrast used | Stable | *-* | *-* |
| 20 | M | 26.6 | Left intraorbital ON | No | Stable | *-* | *-* |
| 21 | F | 32.7 | Right and left prechiasmatic ON, chiasm, right and left radiations | No | Stable | *-* | *-* |
| 22 | F | 3.2 | Right and left intraorbital ON, right and left prechiasmatic ON, chiasm, right and left radiations | Mild | Stable | *-* | *-* |
| 23 | M | 15 | Left intraorbital ON | No | Stable | *-* | *-* |
| 24 | M | 8.8 | Left prechiasmatic ON, chiasm | Avid, no enhancement after regression | Decreased in size | *9.6* | *-* |
| 25 | M | 9.7 | Left intraorbital ON | No contrast used | Stable | *-* | *-* |
| 26 | M | 2.5 | Right intraorbital ON | No | Stable | *-* | *-* |
| 27 | F | 13.1 | Right radiations | Avid, after regression mild enhancement | Decreased in size | *16.9* | *-* |
| 28 | F | 29.9 | Right intraorbital ON, left radiation | No | Stable | *-* | *-* |
| 32 | F | 8.3 | Left prechiasmatic ON | No | Stable | *-* | *-* |
| 34 | M | 9.8 | Chiasm | No | Stable | *-* | *-* |
| 36 | F | 23.7 | Right intraorbital ON | No | Stable | *-* | *-* |
| 37 | F | 4.0 | Left intraorbital ON, left prechiasmatic ON, chiasm | Mild, avid enhancement after progression | Increased in size | *5.0* | *-* |
| 38 | M | 3.8 | Right and left prechiasmatic ON | No contrast used | Stable | *-* | *-* |
| 40 | M | 22.3 | Right prechiasmatic ON | No | Stable | *-* | *-* |
| 42 | F | 35.9 | Left prechiasmatic ON | No | Stable | *-* | *-* |
| 44 | M | 10.5 | Right and left prechiasmatic ON, chiasm | No | Stable | *-* | *-* |
| 46 | F | 4.6 | Left intraorbital ON, left prechiasmatic ON | No | Stable | *-* | *-* |
| 47 | F | 32.9 | Right and left intraorbital ON, left prechiasmatic ON | No | Stable | *-* | *-* |
| 48 | F | 2.5 | Right and left intraorbital ON, left prechiasmatic ON | Diffuse, no enhancement after regression | Stable | *-* | *-* |
| 51 | M | 5.2 | Right and left prechiasmatic ON, chiasm, right and left radiations | No | Stable | *-* | *-* |
| 52 | F | 30.3 | Right prechiasmatic ON | No | Stable | *-* | *-* |
